# Tumour stage and gender predict recurrence and second primary malignancies in head and neck cancer: a multicentre study within the INHANCE consortium

**DOI:** 10.1007/s10654-018-0409-5

**Published:** 2018-05-19

**Authors:** Emanuele Leoncini, Vladimir Vukovic, Gabriella Cadoni, Luca Giraldi, Roberta Pastorino, Dario Arzani, Livia Petrelli, Victor Wünsch-Filho, Tatiana Natasha Toporcov, Raquel Ayub Moyses, Keitaro Matsuo, Cristina Bosetti, Carlo La Vecchia, Diego Serraino, Lorenzo Simonato, Franco Merletti, Paolo Boffetta, Mia Hashibe, Yuan-Chin Amy Lee, Stefania Boccia

**Affiliations:** 1 Section of Hygiene, Institute of Public Health, Università Cattolica del Sacro Cuore, Rome, Italy; 2 Institute of Otorhinolaryngology, Università Cattolica del Sacro Cuore, Rome, Italy; 3 Faculdade de Saúde Pública, University of São Paulo, São Paulo, Brazil; 4 Cirurgia de Cabeça e Pescoço (LIM 28), Faculdade de Medicina, University of São Paulo, São Paulo, Brazil; 5 Aichi Cancer Center Research Institute, Nagoya, Japan; 6 Department of Oncology, IRCCS-Istituto di Ricerche Farmacologiche Mario Negri, Milan, Italy; 7 Department of Clinical Sciences and Community Health, University of Milan, Milan, Italy; 8 SOC Epidemiologia e Biostatistica, IRCCS Centro di Riferimento Oncologico, Aviano, Italy; 9 Laboratory of Public Health and Population Studies, Department of Molecular Medicine, Padua, Italy; 10 Department of Medical Sciences, University of Turin, Turin, Italy; 11 The Tisch Cancer Institute and Institute of Translational Epidemiology, Icahn School of Medicine at Mount Sinai, New York, NY USA; 12 Division of Public Health, Department of Family and Preventive Medicine and Huntsman Cancer Institute, University of Utah School of Medicine, Salt Lake City, UT USA; 13 Division of Public Health, Department of Family and Preventive Medicine, University of Utah School of Medicine, Salt Lake City, UT USA; 14 Section of Hygiene, Institute of Public Health, Università Cattolica del Sacro Cuore, Fondazione Policlinico “Agostino Gemelli” IRCCS, Largo F. Vito, 1, 00168 Rome, Italy

**Keywords:** Head and neck cancer, Preventive, Predictors, Recurrence, Second primary cancer

## Abstract

Recurrence and second primary cancer (SPC) continue to represent major obstacles to long-term survival in head and neck cancer (HNC). Our aim was to evaluate whether established demographics, lifestyle-related risk factors for HNC and clinical data are associated with recurrence and SPC in HNC. We conducted a multicentre study by using data from five studies members of the International Head and Neck Cancer Epidemiology consortium—Milan, Rome, Western Europe, Sao Paulo, and Japan, totalling 4005 HNC cases with a median age of 59 (interquartile range 52–67). Multivariate hazard ratios (HRs) and 95% confidence intervals (CIs) were estimated for recurrence and SPC. During follow-up, 1161 (29%) patients had recurrence and 343 (8.6%) developed SPC. Advanced tumour stage was associated with increased risk of recurrence in HNC overall (HR = 1.76, 95% CI 1.41–2.19). Women with laryngeal cancer had a reduced risk of recurrence compared to men (HR = 0.39, 95% CI: 0.24–0.74). Concerning predictors of SPC, advanced age (HR = 1.02; 95% CI: 1.00–1.04) and alcohol consumption (> 1 drink per day, HR = 2.11; 95% CI: 1.13–3.94) increased the risk of SPC among patients with laryngeal cancer. Additionally, women were at higher risk of SPC, in HNC overall group (HR = 1.68; 95% CI: 1.13–2.51) and oropharyngeal cancer group (HR = 1.74; 95% CI: 1.02–2.98). Tumour stage and male gender (larynx only) were positive predictors of cancer recurrence in HNC patients. Predictors of SPC were advanced age and alcohol use among laryngeal cancer cases, and female gender for oropharyngeal and HNC overall.

## Introduction

Head and neck cancer (HNC) is the sixth most common cancer in men and the 13th in women worldwide, with 195,000 new cases estimated in the South-East Asia Region, 148,000 in the European Region and 101,000 in the Region of the Americas in 2012. These regions account for about three-fourths of worldwide HNC cases [1]. Following diagnosis of HNC, 5-year relative survival varies substantially across countries [2–4]. In Europe, five-year age-standardised relative survival is the highest for laryngeal cancer and the poorest for hypopharyngeal cancer: 59% for larynx, 45% for oral cavity, 39% for oropharynx, and 25% for hypopharynx. For those surviving the first year, the 5-year conditional survival probability increases to 71% for larynx, 62% for oral cavity, 58% for oropharynx and 41% for hypopharynx [3].

Recurrent disease and second primary cancer (SPC) continue to represent the major obstacles to long-term survival in HNC [6, 7]. Despite advances in the treatment of HNC, it is currently well established that the percentage of patients who will develop recurrent disease can be as high as 50% [8]. HNC survivors also have an increased risk of developing SPC compared to the overall population, with frequent SPC of the head and neck, oesophagus, and lung, which are tobacco- and alcohol-related cancers [6]. The incidence of SPC varies substantially across several studies, depending mainly on the follow-up time and systematic screening of patients with HNC [6]. A multicentre study from 13 population-based cancer registries in Europe, Canada, Australia, and Singapore, including 99,257 patients who were diagnosed with a first HNC between 1943 and 2000 found a proportion of SPC development of 10.9% [9].

So far, a few large studies evaluated whether established demographics and lifestyle-related risk factors for HNC influence recurrence, and development of SPC in HNC patients. To explore these issues, we conducted a multicentre study by using data from studies conducted in Brazil, Italy, and Japan, which are members of the International Head and Neck Cancer Epidemiology (INHANCE) Consortium, totalling 4005 HNC cases.

## Methods

### Study population

We included patients 18 years and older with histologically confirmed primary squamous cell carcinoma of the head and neck arising in the four major anatomical sites of the oral cavity, oropharynx, hypopharynx, and larynx. Patients with primary cancers outside these four anatomical sites, patients with a history of previous cancers, and with incomplete data or follow-up were excluded. Participants were selected from five referent studies within the INHANCE Consortium [10]: Milan (Italy), Rome (Italy), Western Europe involving three Italian centres [Aviano (Friuli Venezia Giulia), Padua (Veneto), Turin (Piemonte)], Sao Paulo (Brazil), and Nagoya (Japan).

The study was approved by the local Ethical Committees at each participating centre and written informed consents were obtained from all study subjects. The recruitment period started in 2001 for Nagoya, 2002 for Aviano, Milan, Padua, Rome, and Sao Paulo, and ended in 2005 for Aviano, Padua, Turin and Japan, 2009 for Milan, and 2014 for Rome and Sao Paulo. A total of 4005 eligible subjects were included in the analysis of recurrence, while 3982 subjects were considered for the analysis of SPC.

### Data collection

Patients were interviewed face-to-face in all centres by trained interviewers or medical doctors using a structured and validated questionnaire. All patients were evaluated for gender, age, ethnicity, education level, site of primary tumour, tumour, node, metastasis (TNM) stage, treatment characteristics, comorbidity and smoking and alcohol consumption. Information on alcohol and smoking habits considered the time period ending 1 year prior to HNC diagnosis. These data were pooled and managed by the INHANCE consortium coordinator. Data on tumour pathology, treatment characteristics, cancer recurrence and SPC were obtained from medical records and cancer registries. Different study centres used different cancer codes, which were converted into International Classification of Diseases for Oncology (ICD-O-2) when included into this study.

All cases of first primary HNCs were followed up for cancer recurrence and/or SPC from the date of initial head and neck cancer diagnosis to the date of event, end of follow-up, or loss to follow-up, whichever occurred first. Death certificate data were used to track the cause of death which was coded according to the ICD, Ninth Revision. All the data from participating centres were collected by the project team at the Università Cattolica del Sacro Cuore in Rome and were cleaned and checked for internal consistency. Clarifications were requested from the original investigators when needed.

### Study variables and outcomes definition

HNCs were classified according to the following anatomic sites using the ICD-O-2 codes: oral cavity (C00.3–C00.9, C02.0–C02.3, C03.0, C03.1, C03.9, C04.0, C04.1, C04.8, C04.9, C05.0, C06.0–C06.2, C06.8, and C06.9), oropharynx (C01.9, C02.4, C05.1, C05.2, C09.0, C09.1, C09.8, C09.9, C10.0–C10.4, C10.8, and C10.9), hypopharynx (C12.9, C13.0–C13.2, C13.8, and C13.9), oral cavity or pharynx overlapping or not otherwise specified (C02.8, C02.9, C05.8, C05.9, C14.0, C14.2, and C14.8), larynx (codes C32.0–C32.3 and C32.8–C32.9). Cancers were staged according to the TNM Staging System [11]. Index cancer stage was then dichotomized into early stage (including I and II clinical stage) and late stage (III and IV). We also clustered treatment into five categories: surgery only; surgery with radiation; radiation and/or chemotherapy; surgery plus radiation and/or chemotherapy; and other. Education data were classified into three strata: college graduate, high-technical school graduate and less than high school depending on the level of formal education of participants. Patients were categorized as never, former, or current smokers or missing in case that the information was not available. Cumulative tobacco consumption was calculated as intensity of smoking (never smokers, 20 or less cigarettes/day, > 20 cigarettes/day), and smoking duration in years (never smokers, ≤ 20, > 20 years). Regarding the alcohol drinking status, subjects were classified as never, former and current drinkers, and according to the intensity of use as never drinkers, those who had ≤ 1 drink equivalent/day, and those with > 1 drinks/day. Former users were defined as those who quit cigarette smoking or alcohol consumption for 1 or more years prior to the tumour diagnosis.

Cancer recurrence was defined as local, regional or distant return of cancer of the same histologic type, usually after a period of time during which the cancer could not be detected and after that the patient was defined as disease free. SPC was defined by the criteria of Warren and Gates [12] in the Italian participating centres, where second tumour had to be different in histopathologic type, or in case of the same type to be clearly separated by more than 2 cm of normal epithelium, or occurring more than 3 years after the treatment for the primary tumour. In the Japanese study, SPC was defined as a metachronous invasive solid cancer developing ≥ 6 months after an index HNC. If the second cancer was of different histopathologic type, or if it developed in a different location, it was coded as a SPC. If the second cancer was histopathologically the same and developed in the same region as the index cancer, it was only coded as a SPC if greater than 60 months had passed since the index diagnosis. The Brazilian study considered SPC as a new tumour diagnosed after a primary, in another location and confirmed by anatomical exam. In all participating studies, SPC was needed to be pathologically confirmed as distinct malignancy, with the possibility of metastatic tumour being excluded.

### Statistical analysis

Descriptive analyses were conducted to describe the study population by demographic and known HNC risk factors. The survival after initial tumour development was set as the time interval from the diagnosis of the index tumour to most recent follow-up or the patient’s death. The survival rate was calculated with the Kaplan–Meier method, which was also used to plot the survival curves.

The impact of predictor variables on cancer recurrence and development of SPC was determined using univariate and multivariate analyses. In the initial univariate analyses, epidemiological variables included age in years, ethnicity, sex, body mass index (BMI), education, and smoking and alcohol status, while clinical characteristics included tumour site, stage, and presence of comorbidity. A multivariable proportional hazards model was set up by including the variables that reported a prognostic potential in the univariate analysis (*p* < 0.1). The Cox’s proportional hazards model was used to determine independent predictors of cancer recurrence and SPC. We used Schoenfeld residuals to formally test the Cox proportional hazards assumption for each covariate [13]. Analyses were performed for overall HNC and for separate subsite (oral cavity, oropharynx, hypopharynx and larynx) where possible, and statistical significance was set at *p* < 0.05. We also restricted our study population for the SPC analyses, to the patients with follow-up of at least two years, since it has been reported that the interval between the index and the second tumour ranges from 2 to 4 years [14, 15]. All statistical analyses were performed using Stata software (StataCorp. 2015. Stata Statistical Software: Release 14. College Station, TX: StataCorp LP).

## Results

We included a total of 4005 HNC cases in the recurrence analysis and 3982 cases in the SPC analysis from five studies within the INHANCE consortium (Table 1). The majority of the patients were from Brazil, both for the recurrence (70.7%) and the SPC (70.8%) analysis, then from Italy–Rome, Milan, Aviano, Padua and Turin (21.9% for the recurrence, and 21.8% for SPC), and finally from Japan (7.3 and 7.4% of the cases in recurrence and SPC analyses, respectively).

**Table 1:** Characteristics of head and neck cancer cases from 5 studies participating in the international head and neck cancer epidemiology (INHANCE) Consortium, according to tumour site

INHANCE studies	Recruitment period	Oral cavity		Oropharynx		Hypopharynx		Larynx		OC, OP, HP NOS		Total	
		n	%^a^	n	%^a^	n	%^a^	n	%^a^	n	%	n	%^b^
4005 cases included in recurrence analysis													
Milan, Italy	2002–2009	19	16.2	7	6.0	7	6.0	84	71.8	3	2.5	120	3.0
Rome, Italy	2002–2014	79	18.5	73	17.1	18	4.2	256	60.1	5	1.2	431	10.8
Western Europe													
*Aviano*	2002–2005	40	38.1	32	30.5	8	7.6	25	23.8	2	1.9	107	2.7
*Padua*	2002–2005	24	22.0	24	22.0	14	12.8	47	43.1	1	0.9	110	2.7
*Turin*	2003–2005	47	43.9	22	20.6	7	6.5	31	29.0	3	2.7	110	2.7
Sao Paulo, Brazil	2002–2014	995	38.3	592	22.8	255	9.8	755	29.1	236	8.3	2833	70.7
Japan	2001–2005	147	50.0	49	16.7	47	16.0	51	17.3	0	0.0	294	7.3
Total		1351	36.0	799	21.3	356	9.5	1249	33.3	250	6.2	4005	100.0
3982 cases included in second primary cancer analysis													
Milan, Italy	2002–2009	19	16.2	7	6.0	7	6.0	84	71.8	3	2.5	120	3.0
Rome, Italy	2002–2014	76	18.4	72	17.4	20	4.8	246	59.4	4	1.0	418	10.5
Western Europe													
*Aviano*	2002–2005	42	37.8	34	30.6	9	8.1	26	23.4	2	1.8	113	2.8
*Padua*	2002–2005	25	21.7	25	21.7	14	12.2	51	44.3	1	0.9	116	2.9
*Turin*	2003–2005	44	43.6	21	20.8	5	5.0	31	30.7	2	1.9	103	2.6
Sao Paulo, Brazil	2002–2014	987	38.2	587	22.7	254	9.8	755	29.2	235	8.3	2818	70.8
Japan	2001–2005	147	50.0	49	16.7	47	16.0	51	17.3	0	nc	294	7.4
Total		1340	35.9	795	21.3	356	9.5	1244	33.3	247	6.2	3982	100.0

*OC* oral cavity, *OP* oropharynx, *HP* hypopharynx, *NOS* not otherwise specified, *nc* not computable

^a^Ercentages were calculated excluding OC, OP, HP NOS

^b^Column percentages

The recurrence analysis included 1351 (36.0%) patients with oral cavity cancer, 1249 (33.3%) laryngeal cancer, 799 (21.3%) oropharyngeal cancer, 356 (9.5%) hypopharyngeal cancer, and 250 (6.2%) patients with oral cavity or pharynx not otherwise specified cancer. SPC analysis included 1340 (35.9%) patients with oral cavity cancer, 1244 (33.3%) laryngeal cancer, 795 (21.3%) oropharyngeal cancer, 356 (9.5%) hypopharyngeal cancer and the rest of the patients (6.2%) with oral cavity or pharynx not otherwise specified cancer. Median follow up time for cases included in the recurrence analysis was 21 (IQR 9–55) months, ranged from 17 (IQR 8–40) months in Sao Paulo to 88 (IQR 53–99) months in Aviano. During the follow-up, 1161 (29.0%) patients had cancer recurrence. When looking at the location of primary cancer, during the follow-up 117 (32.9%) hypopharyngeal cancer patients had a recurrence, 423 (31.3%) oral cancer patients, 244 (30.5%) oropharyngeal cancer patients, 296 (23.7%) laryngeal cancer patients and 81 (32.4%) oral cavity or pharynx not otherwise specified cancer cases.

Regarding the 3982 patients included in SPC analysis, median follow-up time was 26 (IQR 11–59) months, from 20 (IQR 10–44) months in the Sao Paulo study to 82 (IQR 31–98) months in Aviano, during which 343 (8.6%) patients developed an SPC. Around 10% of patients with primary laryngeal cancer had SPC, 8.1% of patients with oral cavity cancer, 7.7 and 7.6% with oropharynx and hypopharynx cancer, respectively, and 8.5% of patients with oral cavity or pharynx not otherwise specified cancer developed an SPC (Table 2).

**Table 2:** Median survival time and number of cancer recurrences and second primary cancer by tumour site and study

	Cases included in recurrence analysis (n = 4005)						Cases included in second primary cancer analysis (n = 3982)					
	n	Follow-up time (months)			Recurrence		n	Follow-up time (months)			Second primary cancer	
		Median	1Q	3Q	n	%		Median	1Q	3Q	n	%
Tumour site												
Oral cavity	1351	19	8	50	423	31.3	1340	24	12	55	108	8.1
Oropharynx	799	15	7	40	244	30.5	795	18	9	50	61	7.7
Hypopharynx	356	15	7	34	117	32.9	356	20	10	46	27	7.6
Larynx	1249	30	12	66	296	23.7	1244	37	15	69	126	10.1
OC, OP, HP NOS	250	21	7	63	81	32.4	247	25	10	64	21	8.5
INHANCE studies												
Milan, Italy	120	50	17	77	20	16.7	120	49	17	79	21	17.5
Rome, Italy	431	27	10	63	169	39.2	418	45	17	82	53	12.7
Western Europe												
Aviano	107	88	53	99	21	19.6	113	82	31	98	23	20.4
Padua	110	35	12	96	49	44.5	116	28	8	92	43	37.1
Turin	110	53	11	96	41	37.3	103	60	15	99	18	17.5
Sao Paulo, Brazil	2833	17	8	40	767	27.1	2818	20	10	44	172	6.1
Japan	294	51	14	69	94	32.0	294	59	36	74	13	4.4
Total	4005	21	9	55	1161	29.0	3982	26	11	59	343	8.6

*1Q* first quartile, *3Q* third quartile, *OC* oral cavity, *OP* oropharynx, *HP* hypopharynx, *NOS* not otherwise specified

**Fig. 1:**
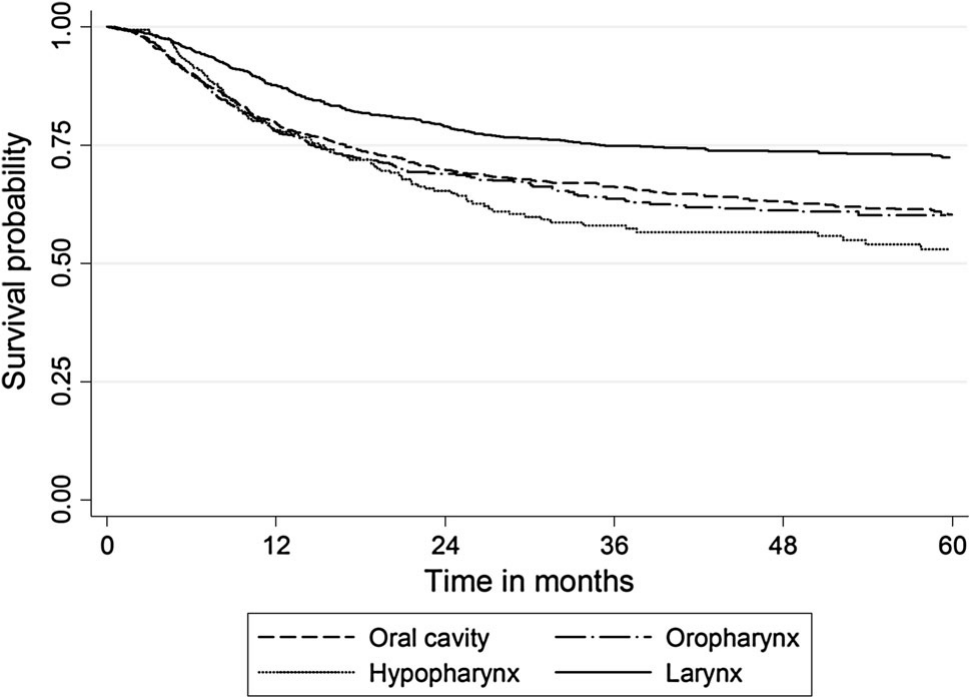
Kaplan–Meier unadjusted recurrence-free 5-year survival by head and neck cancer site

When considering all HNC sites, recurrence-free 5-year survival was 5.90% (standard deviation (SD) = 0.24): oral cavity 6.95% (SD = 0.25), oropharynx 7.30% (SD = 0.26), hypopharynx 3.92% (SD = 0.2), and larynx 4.8% (SD = 0.21) (Fig. 1). On the other hand, SPC-free 5-year survival was 5.97% (SD = 0.24) for all HNC combined, 8.56% (SD = 0.28) for oral cavity, 5.92% (SD = 0.24) oropharynx, 4.92% (SD = 0.22) for hypopharynx and 4.25% (SD = 0.20) for laryngeal cancer (Figure not shown). We further explored the differences in survival by cancer type and for the analysis of the second primary no survival curve was statistically different from the others, while for the recurrence analysis only larynx cancer had a statistically greater survival (*p* < 0.001).

### Predictors of the cancer recurrence

Distributions of the selected covariates and adjusted HRs for cancer recurrence by tumour site and considering HNC overall are presented in Table 3. Median age of patients included in the analysis was 59 years (IQR 52–67) with higher prevalence of males (77.8%), Caucasians (71.9%), normal BMI (57.8%) and low education level (less than high-school, 80.3%). Females with laryngeal cancer had a reduced risk of cancer recurrence (HR = 0.39, 95% CI 0.24–0.74). Tumour stage IV was associated with an increased risk of recurrence in HNC overall (HR = 1.76, 95% CI 1.41–2.19), oral cavity cancer patients (HR = 1.85, 95% CI 1.31–2.61), and oropharyngeal cancer (HR = 2.56, 95% CI 1.19–5.51), while tumour stage III showed to have higher risk of recurrence only in HNC overall (HR = 1.29, 95% CI 1.00–1.67). Exploring the lifestyle habits in detail, we found that patients with oral cavity cancer that were former alcohol consumers had a lower risk of cancer recurrence (HR = 0.64, 95% CI 0.44–0.95) while current consumers with hypopharyngeal cancer had an increased risk (HR = 3.43; 95% CI 1.05–11.26). No further significant association was reported for alcohol drinking and smoking habits.

**Table 3:** Multivariate predictors of cancer recurrence among 4005 head and neck cancer (HNC) cases by tumor site

	Subjects^a^		Oral cavity^b^ (TOT/recurrence)		Oropharynx^b^ (TOT/recurrence)		Hypopharynx^b^ (TOT/recurrence)		Larynx^c^ (TOT/recurrence)		Total^d,e^ (TOT/recurrence)	
			n = 1351/423		n = 799/244		n = 356/117		n = 1249/296		n = 4005/1161	
	n	%	HR	95% CI	HR	95% CI	HR	95% CI	HR	95% CI	HR	95% CI
Demographics												
Age at diagnosis	3990	59 (52–67)^h^	1.00	0.99–1.01	1.01	0.99–1.02	1.00	0.97–1.02	1.00	0.98–1.01	1.00	0.99–1.01
Missing	15	0.4										
Gender												
Men	3104	77.8	1.00		1.00		1.00		1.00		1.00	
Women	887	22.2	0.98	0.76–1.25	0.89	0.63–1.26	1.28	0.75–2.18	**0.39**	**0.24**–**0.74**	0.90	0.76–1.08
Missing	14	0.3										
BMI												
Underweight	192	6.6	1.00		1.00		1.00		1.00		1.00	
Normal range	1679	57.8	0.85	0.54–1.34	1.20	0.60–2.38	0.67	0.26–1.72	1.50	0.65–3.44	0.98	0.71–1.34
Overweight	792	27.3	0.87	0.53–1.41	0.81	0.38–1.73	0.33	0.10–1.04	1.45	0.61–3.43	0.86	0.61–1.20
Obese	243	8.4	0.75	0.41–1.38	0.52	0.18–1.57	0.33	0.10–1.04	1.74	0.68–4.50	0.88	0.59–1.32
Missing	1099	27.4										
Ethnicity												
Caucasian	2830	71.9	1.00		1.00		1.00		1.00		1.00	
Black	236	6.0	0.84	0.55–1.29	0.88	0.43–1.81	0.75	0.23–2.44	0.71	0.34–1.45	0.84	0.61–1.14
Asian	316	8.0	0.84	0.61–1.15	0.67	0.38–1.20	1.64	0.99–2.72	0.40	0.14–1.10	0.81	0.53–1.25
Other	556	14.1	0.84	0.60–1.17	1.38	0.94–2.03	0.92	0.44–1.91	**0.59**	**0.37**–**0.95**	0.89	0.73–1.10
Missing	67	1.7										
Education												
Less than high school	2680	80.3	1.00		1.00		1.00		1.00		1.00	
College/high school graduate	657	19.7	1.16	0.98–1.38	0.73	0.49–1.11	0.74	0.39–1.41	1.36	0.99–1.87	1.19	1.00–1.41
Missing	668	16.7										
Tumour characteristics												
Stage												
I	430	12.9	1.00		1.00		1.00		1.00		1.00	
II	519	15.6	1.22	0.83–1.80	1.62	0.69–3.85	5.20	0.58–46.79	1.09	0.70–1.68	1.24	0.96–1.61
III	561	16.9	1.42	0.95–2.12	2.23	0.99–5.06	2.05	0.27–15.83	1.03	0.66–1.59	**1.29**	**1.00**–**1.67**
IV	1817	54.6	**1.85**	**1.31**–**2.61**	**2.56**	**1.19**–**5.51**	2.81	0.39–20.57	1.37	0.95–1.98	**1.76**	**1.41**–**2.19**
Missing	678	16.9										
Comorbidity												
No	434	52.1	1.00		1.00		1.00		1.00		1.00	
Yes	399	47.9	1.03	0.61–1.75	1.23	0.62–2.45	0.31	0.10–1.04	1.19	0.69–2.05	1.01	0.74–1.37
Missing	3172	79.2										
Cigarette smoking												
Smoking status												
Never	383	9.8	1.00		1.00		1.00		1.00		1.00	
Former	965	24.7	0.72	0.50–1.03	1.16	0.62–2.15	1.15	0.44–3.01	1.37	0.71–2.65	0.87	0.68–1.12
Current	2566	65.6	0.87	0.64–1.18	1.30	0.73–2.31	0.79	0.31–2.04	1.09	0.57–2.08	0.82	0.65–1.04
Missing	91	2.3										
Years of smoking												
Never smokers	383	10.0	1.00		1.00		1.00		1.00		1.00	
≤ 20	384	10.0	0.67	0.41–1.12	1.31	0.66–2.60	0.87	0.28–2.66	1.34	0.65–2.80	0.81	0.60–1.09
> 20	3060	80.0	0.82	0.57–1.18	1.25	0.71–2.20	0.92	0.36–2.34	1.14	0.60–2.16	0.84	0.67–1.06
Missing	178	4.4										
Cigarettes per day												
Never smokers	383	10.0	1.00		1.00		1.00		1.00		1.00	
≤ 20	2430	63.3	0.72	0.59–1.04	1.25	0.71–2.23	0.84	0.33–2.15	1.14	0.60–2.18	0.79	0.63–1.00
> 20	1024	26.7	1.15	0.82–1.62	1.22	0.66–2.23	1.07	0.41–2.83	1.27	0.66–2.45	0.96	0.75–1.22
Missing	168	4.2										
Alcohol drinking												
Drinking status												
Never drinkers	553	14.1	1.00		1.00		1.00		1.00		1.00	
Former	995	25.3	**0.64**	**0.44**–**0.95**	0.99	0.58–1.69	2.45	0.73–8.30	0.98	0.60–1.60	0.88	0.69–1.11
Current	2380	60.6	0.82	0.67–1.27	1.03	0.63–1.69	**3.43**	**1.05**–**11.26**	1.34	0.89–2.02	1.15	0.94–1.41
Missing	77	1.9										
Drinks per day												
Never drinkers	553	15.0	1.00		1.00		1.00		1.00		1.00	
≤ 1	2249	61.1	0.73	0.52–1.02	1.01	0.61–1.68	2.77	0.82–9.39	1.20	0.78–1.85	0.74	0.46–1.09
> 1	877	23.8	1.12	0.79–1.60	1.02	0.60–1.74	3.03	0.89–10.27	1.27	0.82–1.96	0.73	0.49–1.07
Missing	326	8.1										

Text in bold indicates statistically significant risk factors

*CI* confidence interval, *HNC* head and neck cancer, *HR* hazard ratio, *HP* hypopharynx, *nc* not computable, *NOS* not otherwise specified, *OC* oral cavity, *OP* oropharynx

^a^Number of subject and percentages is referred to all HNC sites together

^b^HR adjusted by age at diagnosis, gender and stage

^c^HR adjusted by age at diagnosis, gender, stage, ethnicity and study centre

^d^HR adjusted by age at diagnosis, gender, stage, alcohol drinking status and study centre

^e^Total number includes 250 OC, OP, HP NOS primary HNC with 81 cancer recurrence

^h^Median, interquartile range

### Predictors of the second primary cancer

There were 3081 (77.6%) men and 889 (22.4%) women included in the SPC analysis, with a median age at diagnosis of 59 (IQR, 52–67) years, predominantly Caucasians (71.9%), normal BMI (57.7%) and with low educational level (less than high school, 80.3%). Table 4 presents distribution for the selected covariates and adjusted HRs for SPC by different HNC sites and combined. Female gender was found to increase the risk of developing SPC for those with oropharyngeal cancer (HR = 1.74; 95% CI: 1.02–2.98) and HNC overall (HR = 1.68; 95% CI: 1.13–2.51). Advanced age also increased the risk of SPC development for patients with laryngeal cancer (HR = 1.02; 95% CI: 1.00–1.04). We found lower risk of developing SPC for Asian ethnic group for those with hypopharyngeal (HR = 0.12; 95% CI 0.02–0.91) and laryngeal cancer (HR = 0.04; 95% CI 0.01–0.38). Association was not found for the lifestyle-related risk factors, except the higher risk for those with laryngeal cancer that consumed more than one drink per day (HR = 2.11; 95% CI: 1.13–3.94).

**Table 4:** Multivariate predictors of second primary cancer among 3982 head and neck cancer (HNC) cases by tumor site

	Subjects^a^		Oral Cavity^b^ (TOT/second primary)		Oropharynx^c^ (TOT/second primary)		Hypopharynx^c^ (TOT/second primary)		Larynx^d^ (TOT/second primary)		Total^e,f^ (TOT/second primary)	
			n = 1340/108		n = 795/61		n = 356/27		n = 1244/126		n = 3982/343	
	n	%	HR	95% CI	HR	95% CI	HR	95% CI	HR	95% CI	HR	95% CI
Demographics												
Age at diagnosis	3969	59 (52–67)^g^	1.01	0.97–1.06	1.01	0.99–1.04	0.98	0.94–1.02	**1.02**	**1.00**–**1.04**	1.00	0.98–1.02
Missing	13	0.3										
Gender												
Men	3081	77.6	1.00		1.00		1.00		1.00		1.00	
Women	889	22.4	1.70	0.67–4.30	**1.74**	**1.02**–**2.98**	1.13	0.45–2.84	1.41	0.89–2.22	**1.68**	**1.13**–**2.51**
Missing	12	0.3										
BMI												
Underweight	190	6.6	1.00		1.00		1.00		1.00		1.00	
Normal range	1660	57.7	0.28	0.02–4.83	0.81	0.18–3.58	0.30	0.04–2.65	0.70	0.21–2.31	1.66	0.21–13.44
Overweight	788	27.4	nc	nc	1.00	0.21–4.66	0.83	0.09–7.38	0.62	0.18–2.18	1.08	0.11–10.76
Obese	241	8.4	1.45	0.07–28.14	0.63	0.09–4.52	1.34	0.08–22.14	0.44	0.09–2.23	1.60	0.14–18.17
Missing	1103	27.7										
Ethnicity												
Caucasian	2815	71.9	1.00		1.00		1.00		1.00		1.00	
Black	235	6.0	nc	nc	0.40	0.05–2.92	0.66	0.09–5.01	nc	nc	nc	nc
Asian	316	8.1	nc	nc	0.29	0.07–1.18	**0.12**	**0.02**–**0.91**	**0.04**	**0.01**–**0.38**	nc	nc
Other	550	14.0	1.12	0.13–9.40	0.40	0.12–1.30	0.67	0.19–2.35	0.75	0.32–1.76	1.05	0.29–3.77
Missing	66	1.7										
Education												
Less than high school	2665	80.3	1.00		1.00		1.00		1.00		1.00	
College/high school graduate	653	19.7	0.46	0.10–2.09	0.46	0.20–1.03	0.71	0.24–12.10	0.68	0.41–1.13	0.60	0.35–1.03
Missing	663	16.6										
Tumour characteristics												
Stage												
I–II	933	28.3	1.00		1.00		1.00		1.00		1.00	
III–IV	2367	71.7	2.74	0.97–7.75	1.13	0.57–2.23	nc	nc	0.88	0.55–1.38	1.17	0.72–1.91
Missing	682	17.1										
Comorbidity												
No	428	51.8	1.00		1.00		1.00		1.00		1.00	
Yes	398	48.2	2.38	0.87–6.51	1.09	0.40–2.93	0.20	0.03–1.26	1.13	0.63–2.03	1.14	0.75–1.73
Missing	3156	79.3										
Cigarette smoking												
Smoking status												
Never	386	9.9	1.00		1.00		1.00		1.00		1.00	
Former	952	24.5	1.00	0.27–3.65	0.87	0.33–2.28	0.84	0.16–4.43	1.04	0.43–2.53	0.63	0.33–1.19
Current	2554	65.6	1.84	0.52–6.48	1.30	0.57–3.00	0.80	0.17–3.71	1.75	0.75–4.07	1.22	0.67–2.20
Missing	90	2.3										
Years of smoking												
Never smokers	386	10.1	1.00		1.00		1.00		1.00		1.00	
≤ 20	382	10.0	nc	nc	0.79	0.23–2.75	0.36	0.03–4.15	0.83	0.28–2.50	0.41	0.15–1.12
> 20	3039	79.8	1.59	0.54–4.71	1.23	0.54–2.79	0.87	0.19–3.99	1.54	0.67–3.55	1.00	0.58–1.75
Missing	175	4.4										
Cigarettes per day												
Never smokers	386	10.1	1.00		1.00		1.00		1.00		1.00	
≤ 20	2423	63.5	1.44	0.46–4.47	1.24	0.54–2.85	0.90	0.19–4.19	1.32	0.57–3.07	0.83	0.47–1.48
> 20	1005	26.4	1.22	0.27–5.48	1.00	0.39–2.53	0.60	0.11–3.28	1.74	0.73–4.15	1.12	0.61–2.08
Missing	168	4.2										
Alcohol drinking												
Drinking status												
Never drinkers	548	14.0	1.00		1.00		1.00		1.00		1.00	
Former	996	25.5	8.15	0.87–76.28	1.01	0.38–2.65	1.59	0.33–7.80	1.16	0.44–3.06	1.23	0.55–2.73
Current	2362	60.5	5.07	0.62–41.32	1.44	0.63–3.29	1.09	0.23–5.09	1.19	0.46–3.12	1.30	0.67–2.53
Missing	76	1.9										
Drinks per day												
Never drinkers	548	15.0	1.00		1.00		1.00		1.00		1.00	
≤ 1	2240	61.2	4.42	0.45–43.34	1.08	0.44–2.63	0.77	0.15–3.83	1.58	0.82–3.05	0.65	0.19–2.25
> 1	870	23.8	6.14	0.75–49.99	1.54	0.66–3.62	1.48	0.31–7.15	**2.11**	**1.13**–**3.94**	1.12	0.33–3.75
Missing	324	8.1										

Text in bold indicates statistically significant risk factors

*CI* confidence interval, *HNC* head and neck cancer, *HR* hazard ratio, *HP* hypopharynx, *nc* not computable, *NOS* not otherwise specified, *OC* oral cavity, *OP* oropharynx

^a^Number of subject and percentages is referred to all HNC sites together

^b^HR adjusted by age at diagnosis, gender, stage and comorbidities

^c^HR adjusted by age at diagnosis and gender

^d^HR adjusted by age at diagnosis, gender, number of drinks per day and study centre

^e^HR adjusted by age at diagnosis, gender, ethnicity, education level, comorbidities and alcohol drinking status

^f^Total number includes 247 OC, OP, HP NOS primary HNC with 21 s primary cancer

^g^Median, interquartile range

Exploring the same predictors but considering the specific location of SPC (HNC, lung or other) independently from the type of primary HNC, we included a total of 4021 patients with HNC into this analysis, of whom 113 (32.8%) had HN SPC, 54 (15.7%) had lung SPC and 82 (23.8%) had an SPC in other location, while 95 (27.6%) had no information of SPC site location (Table 5). Analysis also included 39 patients with missing information on primary HNC site, of whom one patient had SPC. They were not considered for the SPC analysis reported in Table 4. Female gender was associated with higher risk of developing a HN SPC (HR = 1.54; 95% CI 1.01–2.35), as well as lung SPC (HR = 4.29; 95% CI 2.24–8.23) and other location SPC (HR = 1.88; 95% CI: 1.13–3.13). Ethnicity and education were found to be associated with lower risk of SPC. We found lower risk for college/high school graduate (HR = 0.59; 95% CI 0.39–0.82) when all cancer sites were combined. Further lower risk for the development of HN SPC was reported for those college/high school graduates (HR = 0.49; 95% CI 0.27–0.91), as well as for patients with Asian ethnicity for the development of other location SPC (HR = 0.11; 95% CI 0.01–0.78). Presence of comorbidity was associated with a higher risk of lung SPC (HR = 3.68; 95% CI 1.04–13.02).

**Table 5:** Multivariate predictors of second primary specific cancer site among 4021 head and neck cancer (HNC) cases

	Subjects^a,b^		Second primary cancer						All second primary cancer sites combined^f,g^ n = 344	
			HNC^c^ n = 113		Lung^d^ n = 54		Other^e^ n = 82			
	n	%	HR	95% CI	HR	95% CI	HR	95% CI	HR	95% CI
Demographics										
Age at diagnosis	4008	59 (52–67)^h^	1.00	0.98–1.02	1.01	0.98–1.04	1.00	0.98–1.02	1.01	1.00–1.02
Missing	13	0.3								
Gender										
Men	3111	77.6	1.00		1.00		1.00		1.00	
Women	898	22.4	**1.54**	**1.01**–**2.35**	**4.29**	**2.24**–**8.23**	**1.88**	**1.13**–**3.13**	1.11	0.82–1.51
Missing	12	0.3								
BMI										
Underweight	194	6.7	1.00		1.00		1.00		1.00	
Normal range	1676	57.6	0.67	0.27–1.71	0.84	0.11–6.57	0.50	0.15–1.70	0.79	0.40–1.58
Overweight	794	27.3	0.46	0.16–1.29	0.91	0.11–7.81	0.52	0.14–1.89	0.65	0.32–1.35
Obese	245	8.4	0.65	0.20–2.14	0.56	0.03–9.02	0.47	0.09–2.34	0.46	0.18–1.13
Missing	1112	27.7								
Ethnicity										
Caucasian	2846	72.0	1.00		1.00		1.00		1.00	
Black	236	6.0	0.74	0.27–2.04	nc	nc	0.28	0.04–2.05	0.47	0.21–1.06
Asian	316	8.0	nc	nc	nc	nc	**0.11**	**0.01**–**0.78**	1.48	0.36–6.06
Other	556	14.1	1.00	0.57–1.78	0.37	0.09–1.54	0.52	0.20–1.30	0.63	0.39–1.01
Missing	67	1.7								
Education										
Less than high school	2665	80.3	1.00		1.00		1.00		1.00	
College/high school graduate	653	19.7	**0.49**	**0.27**–**0.91**	0.52	0.22–1.23	0.70	0.38–1.28	**0.59**	**0.39**–**0.82**
Missing	703	17.5								
Tumour characteristics										
Stage										
I–II	933	28.2	1.00		1.00		1.00		1.00	
III–IV	2374	71.8	1.02	0.65–1.61	1.46	0.71–3.02	0.99	0.57–1.72	1.30	0.98–1.74
Missing	714	17.8								
Comorbidity										
No	440	52.4	1.00		1.00		1.00		1.00	
Yes	400	47.6	0.98	0.46–2.09	**3.68**	**1.04**–**13.02**	1.06	0.52–2.13	1.14	0.75–1.73
Missing	3181	79.1								
Cigarette smoking										
Smoking status										
Never	392	10.0	1.00		1.00		1.00		1.00	
Former	964	24.5	1.23	0.58–2.62	0.77	0.21–2.74	0.53	0.25–1.14	1.05	0.66–1.68
Current	2574	65.5	1.46	0.72–2.94	2.43	0.82–7.72	0.75	0.38–1.47	**1.57**	**1.01**–**2.44**
Missing	91	2.3								
Years of smoking										
Never smokers	392	10.2	1.00		1.00		1.00		1.00	
≤ 20	387	10.1	0.98	0.39–2.45	nc	nc	0.32	0.11–1.02	0.88	0.49–1.59
> 20	3057	79.7	1.49	0.75–2.97	2.20	0.75–6.75	0.73	0.38–1.42	1.44	0.94–2.21
Missing	185	4.6								
Cigarettes per day										
Never smokers	392	10.2	1.00		1.00		1.00		1.00	
≤ 20	2439	63.5	1.32	0.66–2.66	1.56	0.52–4.69	0.69	0.36–1.36	1.28	0.83–1.98
> 20	1011	26.3	1.57	0.74–3.31	2.59	0.83–8.12	0.62	0.29–1.32	1.58	1.00–2.50
Missing	179	4.5								
Alcohol drinking										
Drinking status										
Never drinkers	558	14.1	1.00		1.00		1.00		1.00	
Former	1004	25.4	**3.87**	**1.78**–**8.44**	1.87	0.41–8.46	2.05	0.59–7.11	1.67	0.89–3.14
Current	2383	60.4	**2.24**	**1.06**–**4.74**	1.63	0.36–7.30	2.12	0.62–7.28	1.65	0.89–3.06
Missing	76	1.9								
Drinks per day										
Never drinkers	558	15.1	1.00		1.00		1.00		1.00	
≤ 1	2255	61.2	**6.64**	**2.37**–**18.62**	2.42	0.86–6.85	**2.47**	**1.00**–**6.11**	**1.51**	**1.00**–**2.29**
> 1	872	23.7	**5.78**	**1.94**–**17.25**	**3.38**	**1.29**–**8.84**	**2.88**	**1.21**–**6.87**	1.37	0.89–2.10
Missing	336	8.4								

Text in bold indicates statistically significant risk factors

*CI* confidence interval, *HNC* head and neck cancer, *HR* hazard ratio, *nc* not computable

^a^Number of subject and percentages is referred to all HNC sites together

^b^Total number of subject includes 39 patients with missing information on HNC site

^c^HR adjusted by age at diagnosis, gender and alcohol drinking status

^d^HR adjusted by age at diagnosis, gender and number of drinks per day

^e^HR adjusted by age at diagnosis, gender, ethnicity and number of drinks per day

^f^HR adjusted by age at diagnosis, gender, education level, ethnicity, number of drinks per day and study centre

^g^Total second primary cancer include 95 patients with missing information on second primary cancer site

^h^Median, interquartile range

Alcohol drinking was found to be associated with an increased risk of developing a HN SPC (former drinking status, HR = 3.87; 95% CI 1.78–8.44; current drinking status, HR = 2.24; 95% CI 1.06–4.74; ≤ 1 drinks per day, HR = 6.64, 95% CI 2.37–18.62; > 1 drinks per day HR = 5.78, 95% CI 1.94–17.25), lung SPC (> 1 drinks per day, HR = 3.38, 95% CI 1.29–8.84), and also for other SPC (≤ 1 drinks per day, HR = 2.47, 95% CI 1.00–6.11; > 1 drinks per day HR = 2.88, 95% CI 1.21–6.87). On the other hand, only current smokers had an increased risk of SPC when all SPC sites were combined (HR = 1.57; 95% CI 1.01–2.44) (Table 5).

We also calculated the HRs for the development of SPC after restricting the sample to patients with follow up of at least two years, and found that age was associated with an increased risk of SPC in oral cavity HNC (HR = 1.02, 95% CI 1.00–1.05), drinking status increased the risk in laryngeal cancer (former drinking status, HR = 2.82, 95% CI 1.10–7.23) and all HNC sites combined (HR = 2.08, 95% CI 1.13–3.85). The amount of alcohol consumed was associated with the higher risk of developing SPC in HNC (≤ 1 drinks per day, HR = 3.02, 95% CI 1.27–7.12; > 1 drinks per day, HR = 2.76, 95% CI 1.10–6.92) (data not shown).

## Discussion

In this large multicentre study, we evaluated the prognostic significance of demographic, lifestyle, and clinical characteristics on recurrence and occurrence of SPC. With a median follow-up of 2 years, recurrences occurred in 29% of the study patients, while SPCs in 9%, with the majority in the HNC region followed by cancers of lung. The incidence of recurrences was associated to the primary tumour staging in patients with oral cavity and oropharyngeal cancers. Compared to men, female patients with laryngeal cancer appeared to have a reduced risk of cancer recurrence. With respect to SPCs, female patients with oropharyngeal cancers appeared to have a higher risk than males. Advanced age and high alcohol consumption were risk factor for SPC among patients with laryngeal cancer, while belonging to the Asian ethnic group was protective.

High tumour stage was associated with higher risk of recurrence among patients with oral cavity and oropharyngeal cancers, which is in agreement with other studies [16, 17]. In addition, former alcohol consummation was associated with lower risk of recurrence in patients with oral cavity cancer, which is not in line with our previous study [7], conducted on a smaller sample of patients with cancer recurrence. In this study, we did not investigate established factors associated with recurrence, such as human papillomavirus (HPV) status, and diet [17] because information on these variables was not available. A recent large study examined the prognostic utility of HPV biomarkers among head and neck squamous cell carcinoma cancer across different global regions, using centralized testing and controlling for other risk factors [18]. Tumour p16 and HPV16 DNA positivity were strong biomarkers for improved survival among oropharyngeal squamous cell cancer, but their prognostic utility was not as clear among non-oropharyngeal head and neck squamous cell carcinoma cancer.

Several epidemiologic studies have examined the association between lifestyle habits such as smoking tobacco and consuming alcohol, and SPC in patients with HNC [19, 20]. In our study alcohol, but not tobacco, was a significant risk factor for SPC development. These results are in line with the large body of evidence regarding alcohol intake and SPC risk in patients with HNC. Our previous multicentre study conducted in Italy on 117 HNC patients with SPC reported no significant association for smoking habits, though the risk for developing SPC increased with the increasing years of smoking (> 40) [7]. Subjects from this study were also included in our current analyses [7]. In a recent study including nearly 1000 patients who were treated only in single hospital in South Korea, the reported risk factors for SPC in head and neck squamous-cell carcinoma patients included the index alcohol consumption other than tumour site and patient age [21]. Our results are also consistent with those from a large study in 5 centres from South Europe, which reported a strong association between alcohol consumption and the risk of developing a SPC of the upper aerodigestive tract (UADT) among male patients with laryngeal or hypopharyngeal carcinoma [22]. A systematic review and meta-analysis of existing data from observational studies on the strength of the association of alcohol drinking with SPC risk in patients with UADT reported an increased risk of UADT SPCs, UADT and lung cancers, and for any SPCs [20]. Moreover, two studies investigated the association between SPC and continued alcohol consumption after UADT tumour diagnosis, reporting not consistent results [23, 24]. However, since information on alcohol consumption after diagnosis is unknown, it was not possible to investigate this association in our study. Smoking is the most important risk factor for developing HNC [25, 26]. Thus, it may seem surprising that it does not increase the risk of SPC after HNC as well. We hypothesize that some of the subjects with primary HNC might have stopped using tobacco after diagnosis of the first primary tumour, resulting in a decrease in the risk of tobacco-related SPCs [27]. There is another consideration. Patients who continue smoking after cancer diagnosis experience a consistent increase in risk of death compared with cancer patients who do not smoke after a cancer diagnosis [28], while persistent alcohol use did not affect survival [29].

The most obvious strength of this study was the large number of participants, which included HNC patients from three continents. Due to the large sample size, we were able to evaluate the risk of recurrence or recurrence by HNC subsites adjusting for multiple factors. Another strength is the detailed information about intensity and duration of pre-diagnosis tobacco and alcohol use. Limitations in our study are the lack of information on HPV status when exploring the recurrence of patients with oropharyngeal cancer since the risk of recurrence is lower in HPV-related than HPV-unrelated oropharyngeal squamous cell cancer [18]. Moreover, we did not have data on lifestyle changes after cancer diagnosis over time, which may have affected the prognosis. It was also not possible to investigate the influence of the persistence of tobacco and alcohol use in the appearance of SPC. Lastly, short follow-up period is another limitation, as one participating study had a median follow-up time lower than 2 years, though approximately 35 to 55% of patients with HNC use to experience locoregional recurrence or distant metastasis within 2 years of initial diagnosis [8, 30, 31].

In summary, this large epidemiologic study investigated the association between demographics and lifestyle-related risk factors for HNC with recurrence and SPC development in HNC patients across continents. Tumour stage (both for oral cavity and oropharynx) and male gender (larynx only) were positive predictors of cancer recurrence in HNC patients. Predictors of SPC were advanced age, Asian ethnicity and alcohol among laryngeal cancer cases, and female gender for oropharyngeal and HNC overall. It would be important to collect information on tobacco use, alcohol consumption, diet habits, and other exposures in the interval between the first tumour and the occurrence of recurrence or SPC, to better define their role in reducing cancer recurrence or SPCs.
